# Supplementary material for: Multi‐dataset identification of innovative feature genes and molecular mechanisms in keratoconus
Source: J Cell Mol Med. 2024 Sep 19;28(18):e70079. doi: 10.1111/jcmm.70079 (PMC11412914; doi:10.1111/jcmm.70079)
Supplement: Supplementary file 2 — Material S2. [file JCMM-28-e70079-s002.zip › 7.GSEA analysis/1.ARL11/KEGG_ANTIGEN_PROCESSING_AND_PRESENTATION.html]

Details for gene set KEGG\_ANTIGEN\_PROCESSING\_AND\_PRESENTATION[GSEA]

|  || Dataset | ARL11.group.cls#h\_versus\_l |
| Phenotype | group.cls#h\_versus\_l |
| Upregulated in class | h |
| GeneSet | KEGG\_ANTIGEN\_PROCESSING\_AND\_PRESENTATION |
| Enrichment Score (ES) | 0.27126428 |
| Normalized Enrichment Score (NES) | 0.8903963 |
| Nominal p-value | 0.606 |
| FDR q-value | 1.0 |
| FWER p-Value | 1.0 |
Table: GSEA Results Summary

  

Fig 1: Enrichment plot: KEGG\_ANTIGEN\_PROCESSING\_AND\_PRESENTATION      
 Profile of the Running ES Score & Positions of GeneSet Members on the Rank Ordered List

  

| SYMBOL | TITLE | RANK IN GENE LIST | RANK METRIC SCORE | RUNNING ES | CORE ENRICHMENT || 1 | CD8B | na | 580 | 0.460 | 0.0400 | Yes |
| 2 | CIITA | na | 1433 | 0.343 | 0.0578 | Yes |
| 3 | KIR2DS4 | na | 1594 | 0.329 | 0.0939 | Yes |
| 4 | LTA | na | 2268 | 0.278 | 0.1088 | Yes |
| 5 | CD74 | na | 2423 | 0.269 | 0.1376 | Yes |
| 6 | HLA-DRB5 | na | 2778 | 0.251 | 0.1584 | Yes |
| 7 | IFNA6 | na | 3473 | 0.220 | 0.1656 | Yes |
| 8 | KLRC4 | na | 3556 | 0.217 | 0.1900 | Yes |
| 9 | HLA-DOA | na | 3812 | 0.207 | 0.2083 | Yes |
| 10 | IFNA14 | na | 4269 | 0.191 | 0.2187 | Yes |
| 11 | KIR2DL4 | na | 4763 | 0.173 | 0.2258 | Yes |
| 12 | IFNA16 | na | 4914 | 0.169 | 0.2423 | Yes |
| 13 | HSPA2 | na | 5536 | 0.150 | 0.2430 | Yes |
| 14 | IFNA4 | na | 6226 | 0.131 | 0.2392 | Yes |
| 15 | KIR3DL3 | na | 6575 | 0.122 | 0.2443 | Yes |
| 16 | HLA-DRB4 | na | 7218 | 0.107 | 0.2390 | Yes |
| 17 | HSPA8 | na | 7386 | 0.104 | 0.2470 | Yes |
| 18 | HLA-DRA | na | 7656 | 0.099 | 0.2514 | Yes |
| 19 | HLA-DPA1 | na | 7767 | 0.097 | 0.2602 | Yes |
| 20 | HLA-DRB1 | na | 7965 | 0.093 | 0.2660 | Yes |
| 21 | HLA-DQB1 | na | 8352 | 0.086 | 0.2654 | Yes |
| 22 | CD8A | na | 8817 | 0.078 | 0.2616 | Yes |
| 23 | HSPA1A | na | 9156 | 0.072 | 0.2608 | Yes |
| 24 | B2M | na | 9334 | 0.070 | 0.2643 | Yes |
| 25 | HSPA6 | na | 9438 | 0.068 | 0.2698 | Yes |
| 26 | IFI30 | na | 9665 | 0.065 | 0.2713 | Yes |
| 27 | HLA-F | na | 10625 | 0.052 | 0.2500 | No |
| 28 | CTSB | na | 10717 | 0.051 | 0.2537 | No |
| 29 | HSPA1B | na | 10821 | 0.050 | 0.2568 | No |
| 30 | KLRD1 | na | 10973 | 0.048 | 0.2584 | No |
| 31 | CD4 | na | 11083 | 0.047 | 0.2610 | No |
| 32 | HSP90AA1 | na | 11158 | 0.046 | 0.2645 | No |
| 33 | KIR3DL2 | na | 11581 | 0.041 | 0.2573 | No |
| 34 | TAPBP | na | 12783 | 0.029 | 0.2262 | No |
| 35 | RFXAP | na | 13286 | 0.025 | 0.2148 | No |
| 36 | PSME2 | na | 13573 | 0.022 | 0.2093 | No |
| 37 | CTSS | na | 13667 | 0.021 | 0.2092 | No |
| 38 | HLA-G | na | 14146 | 0.018 | 0.1976 | No |
| 39 | HSPA4 | na | 14351 | 0.016 | 0.1937 | No |
| 40 | HLA-DPB1 | na | 14439 | 0.016 | 0.1931 | No |
| 41 | HLA-E | na | 14680 | 0.014 | 0.1879 | No |
| 42 | CALR | na | 14744 | 0.013 | 0.1877 | No |
| 43 | HSP90AB1 | na | 14766 | 0.013 | 0.1887 | No |
| 44 | KIR2DL5A | na | 14923 | 0.012 | 0.1856 | No |
| 45 | RFXANK | na | 14977 | 0.011 | 0.1855 | No |
| 46 | IFNA2 | na | 15087 | 0.011 | 0.1837 | No |
| 47 | CTSL | na | 15220 | 0.010 | 0.1810 | No |
| 48 | NFYA | na | 15305 | 0.009 | 0.1797 | No |
| 49 | PSME3 | na | 15443 | 0.008 | 0.1767 | No |
| 50 | PSME1 | na | 15451 | 0.008 | 0.1775 | No |
| 51 | KIR2DS3 | na | 15707 | 0.006 | 0.1709 | No |
| 52 | HLA-C | na | 15719 | 0.006 | 0.1713 | No |
| 53 | HSPA1L | na | 16824 | -0.002 | 0.1397 | No |
| 54 | TAP2 | na | 16908 | -0.003 | 0.1376 | No |
| 55 | HLA-DMA | na | 17342 | -0.006 | 0.1258 | No |
| 56 | HLA-DOB | na | 17625 | -0.008 | 0.1186 | No |
| 57 | HLA-A | na | 17972 | -0.010 | 0.1099 | No |
| 58 | HSPA5 | na | 18392 | -0.013 | 0.0994 | No |
| 59 | NFYB | na | 18876 | -0.016 | 0.0874 | No |
| 60 | PDIA3 | na | 18907 | -0.016 | 0.0886 | No |
| 61 | CANX | na | 19174 | -0.018 | 0.0831 | No |
| 62 | HLA-B | na | 19514 | -0.021 | 0.0759 | No |
| 63 | HLA-DRB3 | na | 19647 | -0.021 | 0.0747 | No |
| 64 | TAP1 | na | 20591 | -0.028 | 0.0510 | No |
| 65 | NFYC | na | 21292 | -0.034 | 0.0349 | No |
| 66 | KIR2DL2 | na | 21305 | -0.034 | 0.0387 | No |
| 67 | RFX5 | na | 22026 | -0.040 | 0.0229 | No |
| 68 | HLA-DQA2 | na | 24037 | -0.063 | -0.0274 | No |
| 69 | LGMN | na | 27101 | -0.110 | -0.1022 | No |
| 70 | CREB1 | na | 27227 | -0.113 | -0.0918 | No |
| 71 | HLA-DMB | na | 27501 | -0.119 | -0.0850 | No |
| 72 | KLRC1 | na | 28036 | -0.132 | -0.0841 | No |
| 73 | KLRC3 | na | 28687 | -0.149 | -0.0846 | No |
| 74 | HLA-DQA1 | na | 30949 | -0.219 | -0.1228 | No |
| 75 | IFNA8 | na | 31555 | -0.242 | -0.1104 | No |
| 76 | IFNA10 | na | 33230 | -0.339 | -0.1168 | No |
| 77 | IFNA7 | na | 33254 | -0.341 | -0.0754 | No |
| 78 | IFNA21 | na | 34187 | -0.451 | -0.0466 | No |
| 79 | KIR3DL1 | na | 34421 | -0.504 | 0.0089 | No |
Table: GSEA details [plain text format]

  

Fig 2: KEGG\_ANTIGEN\_PROCESSING\_AND\_PRESENTATION      
 Blue-Pink O' Gram in the Space of the Analyzed GeneSet

  

Fig 3: KEGG\_ANTIGEN\_PROCESSING\_AND\_PRESENTATION: Random ES distribution      
 Gene set null distribution of ES for **KEGG\_ANTIGEN\_PROCESSING\_AND\_PRESENTATION**
